# Liver Abscess and Pseudotumoral Gastric Lesion Caused by Chicken Bone Perforation: Laparoscopic Management

**DOI:** 10.1155/2012/791857

**Published:** 2012-11-06

**Authors:** Gabriele Ricci, Nello Campisi, Giovanni Capuano, Luigi De Vido, Luca Lazzaro, Giuliana Simonatto, Barbara Termini, Valeria Turriziani, Francesco Fidanza

**Affiliations:** Division of General and Laparoscopic Surgery, San Tommaso dei Battuti Hospital, Via Zappetti 58, 30026 Portogruaro, Italy

## Abstract

The accidental ingestion of a foreign body into the gastrointestinal tract is not uncommon, but the vast majority of foreign bodies pass through the gastrointestinal tract uneventfully within a week. Less than 1% of patients with foreign body ingestion develop complications such as perforation of the gastrointestinal tract. The migration of an ingested foreign body may result in chronic inflammation, a silent clinical course, and radiological features suggestive of a neoplasm. The authors report a case of chicken bone perforation of the gastric wall that leads to hepatic abscess formation and thereafter to submucosal pseudotumor of the gastric wall treated with totally laparoscopic management. Usefulness of endoscopic ultrasonography for a correct diagnosis is also stressed.

## 1. Introduction

The accidental ingestion of a foreign body into the gastrointestinal tract is not uncommon, but the vast majority of foreign bodies pass through the gastrointestinal tract uneventfully within a week [1]. Less than 1% of patient with foreign body ingestion develop complications such as perforation of the gastrointestinal tract [2, 3]. Commonly ingested foreign bodies vary by country and depends on dietary habits. Nearly two-thirds of foreign bodies that are causes of complications are fish bones, other examples include toothpicks, shells, and chicken bones [2]. 

The patients in these cases tend not to recall the specifics of the ingestion. In the absence of a reliable history, the migration of an ingested foreign body may result in chronic inflammation, a silent clinical course, and radiological features suggestive of a neoplasm. Herein the authors report a case of chicken bone perforation of the gastric wall that lead to hepatic abscess formation and thereafter to submucosal pseudotumor of the gastric wall treated with totally laparoscopic management. 

## 2. Case Report

**Figure 1:**
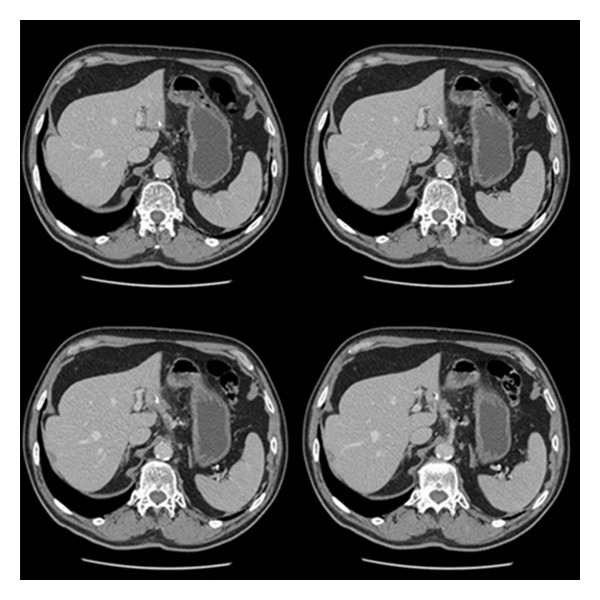
CT scan: presence of a linear radiopaque structure 3 cm in length at the inferior margin of left lobe of the liver.

A 59-year-old previously fit and healthy man was admitted in another hospital with 2-weeks history of intermittent fever with no other symptoms associated. Liver ultrasound revealed the presence of left lobe liver mass 3,2 cm in maximum diameter, suggestive for abscess. The patient was treated with success with long-term antibiotic therapy. Follow-up CT scan of the liver performed at the end of antibiotic treatment revealed the disappearance of liver mass, a linear radiopaque structure 3 cm in length was noted at the inferior margin of left lobe of the liver. No further investigations were done for it because it was interpreted as residual calcification of liver abscess (Figure 1).

One year later the patient presented to the emergency department of our hospital complaining of progressive dysphagia to solids and liquids, associated with weight loss, and cramp-like epigastric pain exacerbated by meals. On examination the patient was apyrexial and haemodynamically stable, abdominal examination was unremarkable. Blood tests revealed a moderate sideropenic anemia: white blood cell count was 9.200/*μ*L, hemoglobin concentration 11,7 g/dL, hematocrit 33,4%, and platelet count 253 × 10^3^/*μ*L. His serum urea nitrogen concentration was 57 mg/dL, creatinine 0,9 mg/dL, AST 14 IU/l, ALT 11 IU/l, Iron 26 mcg/dL, bilirubin, albumin, and other blood component chemestries were all within the normal range. A severe increase in erythrocyte sedimentation rate (106 mm/hr) was also noted. 

**Figure 2:**
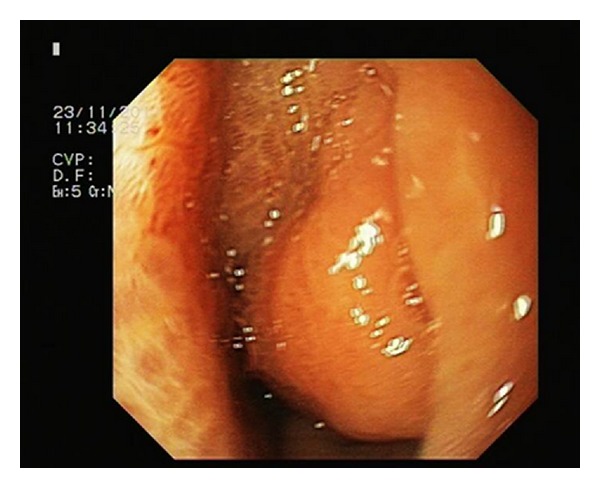
Gastroscopy: presence of a submucosal antral mass causing extrinsic compression on the gastric lumen without a discrete mucosal lesion.

**Figure 3:**
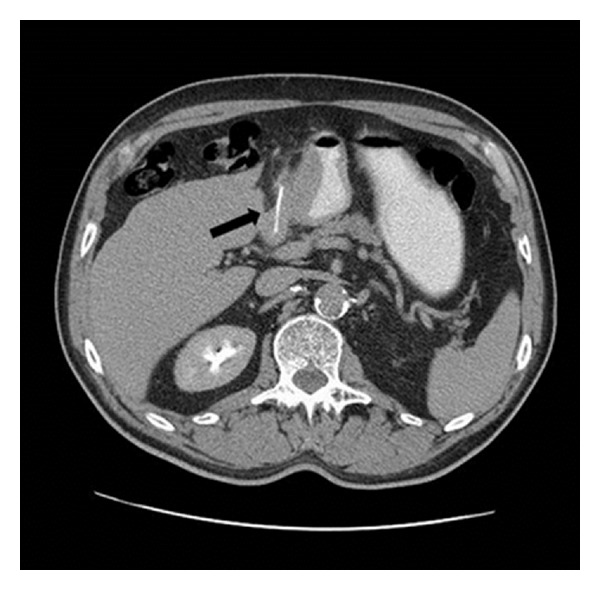
CT scan: submucosal heterogeneous enhancing mass in the distal stomach, that produced a stricture of the gastric lumen. *Black arrow* shows the presence of linear radiopaque foreign body.

**Figure 4:**
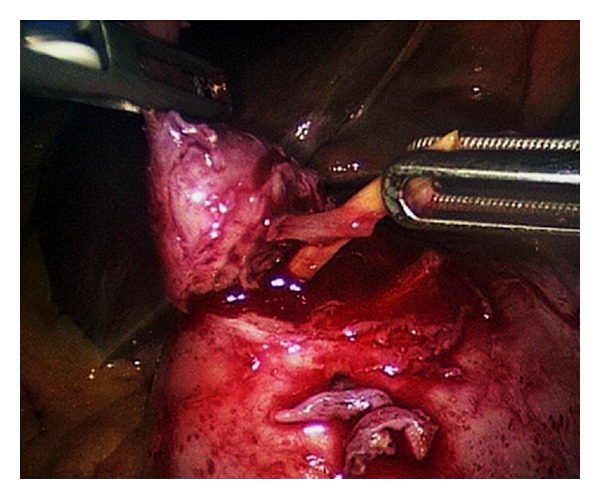
Laparoscopy: extraction of the foreign body from gastric wall.

Upper endoscopy showed a submucosal antral mass causing extrinsic compression on the gastric lumen without a discrete mucosal lesion (Figure 2). Biopsies of the lesion were inconclusive, revealing mild inflammatory changes. CT scan confirmed the presence of a submucosal ill-defined heterogeneous enhancing mass in the distal stomach, that produced a stricture of the gastric lumen (Figure 3). Reactive aorto-cava and celiac axis lymph nodes 1 cm in diameter were also noted. The radiologist did not offer a differential diagnosis, but concluded for a productive lesion of distal gastric wall consistent with the diagnosis of GIST. For this reason endoscopic ultrasound was obtained; narrowing of distal gastric lumen due to submucosal mass was confirmed, moreover in that examination some spillage of white-thick fluid from prepyloric mucosa was noted after compression of the mass. EUS described an heterogeneous hypoechogenic mass 3 cm in maximum diameter with anecogenic central area, consistent with the presence of a foreign body. Explorative laparoscopy was performed; following lysis of adhesions between the inferior surface of the left lobe of the liver and the distal lesser curve of the stomach, a perforation of the gastric wall covered by omentum was noted. The lesion was carefully opened and following drainage of thick-liquid pus material, a foreign body 3 cm in length was identified and extracted from the abscessual cavity (Figure 4). The wall of the abscess was than completely removed and gastric discontinuity was sutured with single layer interrupted vicryl stitches. At the end of procedure fibrin sealant was applied. Definitive pathology report was consistent with fibrous tissue and smooth muscle with chronic inflammatory infiltration, foreign body was demonstrated to be a fragment of chicken bone. Postoperative course was uneventful and patient was discharged in good clinical conditions on 6th postoperative day. 

## 3. Discussion

Unintentional foreign bodies ingested in adults are usually dietary as in the case reported. The number of occasions on which foreign bodies are incidentally ingested are numerous, but the vast majority of foreign bodies pass through the gastrointestinal tract uneventfully within a week [1]. Gastrointestinal tract perforation is rare, occurring in less than 1% of these patients [2, 3]. If the objects are long, hard, and sharp the risk of perforation of gastrointestinal wall is higher. Nearly two-thirds of foreign bodies that are causes of complications are fish bones other examples include toothpicks, shells, and chicken bones [2].

Intra-abdominal foreign body perforations of the gastrointestinal tract have been reported in all segments, although it tends to occur in regions of acute angulation such as the ileocecum, rectosigmoid, or proximally to a site of pathologic narrowing or obstruction [2, 4, 5]. Foreign body perforation of the stomach is quite rare. In the recent series reported by Goh et al. gastric perforation has been reported in 14% of foreign body perforations [2]. 

A foreign body that perforates the bowel wall may take several possible courses, including lying in the bowel lumen at the site of perforation or passing through the gastrointestinal wall to migrate to a distal organ [6]. For this reason intra-abdominal foreign body perforations of the gastroinetstinal tract have a wide spectrum of clinical presentations that may be acute or chronic. They may be classified as acute peritonitis, which may be localized or generalized, an abdominal wall tumor or abscess, or an intra-abdominal mass or abscess formation [2, 7].

Patients with foreign body perforation in the jejunum and ileum typically exhibit an acute onset of symptoms due to localized of generalized peritonitis. On the contrary patients with foreign body perforation in the stomach, duodenum, and large bowel are more likely to present with longer, more innocuous clinical picture, exhibiting chronic symptoms such as abdominal mass and abscess. As postulated by Goh et al. it is possible that a thicker gut wall (stomach and large bowel) causes the foreign body to perforate more gradually, and the close proximity of the omentum and adjacent organs such as the liver assist in “sealing” the perforation site [2]. For this reason there may be a considerable time lag of months or even years between the time of ingestion and the onset of symptoms as in the case reported. Gastric and duodenal perforation may result in rarely reported cases of foreign body-induced hepatic abscess formation [8]. 

If perforating foreign bodies are identified early, namely, in the absence of peritonism, endoscopic retrieval may be possible. In two cases of gastric perforation by a chicken bone without peritoneal irritation, endoscopic extraction and clipping has been described [6, 9]. Unluckily early detection of foreign body ingestion is very rare. 

In the diagnosis of nonmetallic foreign bodies plain radiography is unreliable, even with bony radiopacity, because of the masking effect of the soft tissue mass, fluid collection around the penetrated bone, and the absence of free gas in the abdomen [5, 10]. Free pneumoperitoneum indeed is rare, as the foreign body is gradually impacted and the perforation is covered with fibrin [2, 11]. CT scan is preferred and will usually demonstrate a linear calcified lesion, however the accuracy of CT is limited by the lack of observer awareness, and a high index of suspicion must be maintained for the correct diagnosis [12, 13]. Usually the linear calcified lesion is initially missed and can be seen only in the retrospect. Moreover carcinoid tumors and GISTs may both exhibit flecks of calcification on CT appearance [14].

Definitive history of foreign body ingestion could be obtained preoperatively in very rare cases, and unaware of the ingestion history, clinicians and radiologists placed priority on malignant necrotic lesions. In the case reported from authors the linear calcified lesion was visible on CT scan, but its presence was initially missed by radiologist and it was seen only in the retrospect. Submucosal prepyloric mass was initially considered as a tumor lesion, most probably a GIST. In similar cases reported in the literature preoperative assessment failed to identify the foreign body and these lesions were treated as malignant tumors: Cho et al. reported a pseudotumor of the gastrocolic ligament area due to migration of the ingested crab-leg that was treated with antrectomy and transverse colon wedge resection [11]; Al-Deeb et al. and Bajwa et al. described a similar cases in which a subtotal gastrectomy was performed for a psuedotumoral gastric lesion caused by fish bone perforation [15, 16]; Goh et al. described a case of fish bone perforation of the stomach causing a mass in the pancreatic head area that was treated with a subtotal pancreatectomy, partial gastrectomy, splenectomy, and segmental colectomy [17]; Rao et al. performed a laparotomy with the intent of pancreatoduodenectomy in a patient with pancreatic mass secondary to chicken bone penetration [18]. None of the previous similar cases reported in the literature were managed laparoscopically. 

Chiang et al. reported a case of duodenal perforation caused by a toothpick and complicated by liver abscess controlled successfully with antibiotics, in which endoscopic ultrasonography allowed to clarify the diagnosis of foreign body perforation [19]. Similarly Huang et al. described a case of fish bone-induced submucosal tumor of the gastric wall, in which endoscopic ultrasonography identified the linear calcified fish bone embedded in a heterogeneous mass [20]. In the case reported by the authors the patient was at first successfully treated with antibiotic therapy for a liver abscess, that the internist suspected to be secondary to human brucellosis. The foreign body was not recognized and after one year the patient came to our hospital for dysphagia and weight loss due to prepyloric submucosal gastric mass. On the base of endoscopic and CT scan results we interpreted the mass as a malignant tumor, possibly a GIST. The aid of endoscopic ultrasonography was fundamental because it oriented the diagnosis to inflammatory mass with central abscess secondary to the presence of suspect foreign body. It allowed the correct management of the disease, avoiding huge resection of organs as reported in previous similar cases.
